# Mortality along the continuum of HIV care in Rwanda: a model-based analysis

**DOI:** 10.1186/s12879-016-2052-7

**Published:** 2016-12-01

**Authors:** Eran Bendavid, David Stauffer, Eric Remera, Sabin Nsanzimana, Steve Kanters, Edward J. Mills

**Affiliations:** 1 Division of General Medical Disciplines, Stanford University, Stanford, CA USA; 2 Center for Health Policy and the Center for Primary Care and Outcomes Research, Stanford University, Stanford, CA USA; 3 Institute of HIV Disease Prevention and Control, Rwanda Bio-Medical Center, Kigali, Rwanda; 4 Basel Institute for Clinical Epidemiology & Biostatistics and Swiss Tropical and Public Health Institute, University of Basel, Basel, Switzerland; 5 Precision Global Health, Vancouver, Canada

**Keywords:** HIV, Rwanda, Mortality, Care continuum, Care cascade, Antiretroviral coverage, Universal test and treat, Loss from care

## Abstract

**Background:**

HIV is the leading cause of death among adults in sub-Saharan Africa. However, mortality along the HIV care continuum is poorly described. We combine demographic, epidemiologic, and health services data to estimate where are people with HIV dying along Rwanda’s care continuum.

**Methods:**

We calibrated an age-structured HIV disease and transmission stochastic simulation model to the epidemic in Rwanda. We estimate mortality among HIV-infected individuals in the following states: untested, tested without establishing care in an antiretroviral therapy (ART) program (unlinked), in care before initiating ART (pre-ART), lost to follow-up (LTFU) following ART initiation, and retained in active ART care. We estimated mortality among people living with HIV in Rwanda through 2025 under current conditions, and with improvements to the HIV care continuum.

**Results:**

In 2014, the greatest portion of deaths occurred among those untested (35.4%), followed by those on ART (34.1%), reflecting the large increase in the population on ART. Deaths among those LTFU made up 11.8% of all deaths among HIV-infected individuals in 2014, and in the base case this portion increased to 18.8% in 2025, while the contribution to mortality declined among those untested, unlinked, and in pre-ART. In our model only combined improvements to multiple aspects of the HIV care continuum were projected to reduce the total number of deaths among those with HIV, estimated at 8177 in 2014, rising to 10,659 in the base case, and declining to 5,691 with combined improvements in 2025.

**Conclusion:**

Mortality among those untested for HIV contributes a declining portion of deaths among HIV-infected individuals in Rwanda, but the portion of deaths among those LTFU is expected to increase the most over the next decade. Combined improvements to the HIV care continuum might be needed to reduce the number of deaths among those with HIV.

**Electronic supplementary material:**

The online version of this article (doi:10.1186/s12879-016-2052-7) contains supplementary material, which is available to authorized users.

## Background

The number of HIV infected individuals receiving antiretroviral therapy (ART) in Sub-Saharan Africa is steadily increasing [1]. The expansion of ART provision has led to important declines in HIV-related and all-cause adult mortality [2–4]. However, the declining global estimates of HIV mortality belie the fact that, in 2013, HIV was responsible for more deaths than any other single cause in sub-Saharan Africa [5, 6]. A major challenge for addressing mortality among those with HIV is the incomplete understanding of what patient groups are at the highest risk of mortality. This understanding can inform the prioritization of programs targeting at-risk populations. We aimed to use data from the Rwandan HIV population to estimate mortality along the continuum of care.

The HIV care continuum, sometimes referred to as the care cascade, is a paradigm for understanding the states in the health care system that HIV-infected individuals find themselves following HIV infection [7]. Estimates of the continuum in the United States, for example, suggest that only 30% of all HIV-positive patients were virally suppressed in 2011, with notable gaps prior to HIV testing, after testing and before ART initiation, and after ART initiation [8]. However, healthcare systems are poorly equipped to monitor the vital status of patients outside ART programs, and may fail to distinguish between patient loss from care, transition to other healthcare facilities, and death [9]. As Rwanda’s public health system ensures that most patient transfers and mortality are identified, a unique opportunity exists to assess patient tracking through the HIV care continuum [10]. We used nationally representative data from Rwanda and modeled this using a transmission simulation model.

## Methods

### Setting

Rwanda’s HIV/AIDS strategy has been a positive outlier in its ability to engage large segments of the population, including typically hard to service populations, such as men and adolescents [11]. Since 2002, a National Strategic Plan set aggressive targets for increasing ART coverage using a decentralized network of clinics principally staffed by community health workers [12]. Rwanda has outpaced many other countries in increasing ART coverage (defined as the portion of all infected individuals that are receiving ART) [1]. In addition, nearly all care in Rwanda is provided by the public sector (less than 1% of patients attend private clinics according to national surveillance data) [13].

### Model description

We calibrated a dynamic stochastic microsimulation model of HIV disease and transmission to Rwanda’s HIV epidemic to study where in the HIV care continuum are people dying [14–17]. The model uses information on the transition of patients through the health care system from a national source of HIV care in the public sector [10]. We used the model to estimate the distribution and trends of mortality. Specifically, we examined where along the HIV care continuum are HIV-infected individuals dying, and how this trend is expected to change in the next decade. We also examined how HIV treatment and prevention programs addressing different aspects of HIV care delivery would be expected to affect future mortality patterns.

The model simulates HIV transmission, testing, treatment, and disease progression in individuals, using an age- and gender-stratified demographic model fit to Rwanda’s population [18]. We modeled a population ages 15 and older, and assumed a stable population growth rate of 2.6% per year [18, 19]. The model is calibrated to several epidemiologic trends from 2003 to 2014, including population size, HIV testing coverage, ART coverage, and HIV prevalence [1, 10, 20, 21]. Complete model parameters, description, and calibration figures are shown in Additional file 1: Sections S1 and S2.

We modeled disease transmission through heterosexual contact. The infection status of HIV-negative individuals is determined by their risk factors for acquiring HIV, including the number of sexual partners, the HIV status of their sexual partners, the infectivity of HIV-positive partners, and, for men, their circumcision status. Partner distribution and infectivity based on viral load is shown in the parameter table. The initial distribution of sexual partners was estimated from Rwanda’s 2005 Demographic and Health Survey and updated through discussion with Ministry of Health participation [21]. The probability of having an HIV-infected sexual partner is dynamically determined in relation to the HIV prevalence in the opposite gender. We assume homogenous mixing across ages, and did not explicitly model special populations such as female sex workers except through the distribution of sexual partnerships. For each individual with an HIV-positive partner, the partner’s infectivity is determined by the distribution of acutely infected, unsuppressed, and suppressed individuals in the currently infected population. We assume that individuals with suppressed viral load on ART have 90% lower risk of transmitting HIV per coital act compared with chronic unsuppressed infection (the reduction in infectivity is higher in comparison with acute HIV) [22, 23]. The probability of infection for an uninfected individual, then, is a product of their partners’ infection status and the transmission hazard per person-month of contact given the partner’s infectivity. The risk of acquiring HIV is 50% lower for circumcised men [24, 25]. Additional demographic and transmission parameters are provided in Table 1 and Additional file 1: Section S1.

**Table 1:** Selected model parameters and base case assumptions

Parameter		Base case value	Uncertainty	Source(s)
*Demographic parameters*				
Life expectancy, 2003				
	Male	50.9		World Population Prospects [20]
	Female	52.9		World Population Prospects [20]
Male circumcision prevalence		30%	[20–40]	Binagwaho et al. [33]
Number of sexual partnerships in 12-month period				2010 Rwanda DHS [21]
0		56%	+/− 19%	
1		36%	+/− 9%	
2		7%	+/− 2%	
3+		1%	+/− 0.25%	
*HIV program parameters*				
HIV testing: annual probability of receiving HIV test				
	2003–2010	20%	10–30%	Rwanda DHS [21]
	2010–2014	40%	35–45%	Nsanzimana [10]
Portion of those tested who are connected to care				
	2003–2010	70%		Nsanzimana [10]
	2010–2014	90%		Nsanzimana [10]
Rate of loss from pre-ART care		0.5% monthly	0.3–0.7%	Nsanzimana [10]
ART initiation threshold (CD_4_ counts, cells/mm^3^)				
	2003–2008	250		Nsanzimana [10] and NSP [34]
	2008–2014	350		
	2014	500		
Rate of loss to follow-up from ART care		0.0875% monthly	0.05–0.125%	Nsanzimana [10]
Probability of return after loss from ART care		25%	20–30%	Nsanzimana [10]
*Morbidity & mortality parameters*				
Age-specific mortality	Yearly	Age-specific _n_q_x_		WHO life tables [28]
CD4-specific mortality	6 CD4 bins	Mortality rate by CD4	95% CI for each rate	Nsanzimana [30], table 2
OD-specific risk by CD4	6 CD4 bins	Mortality rate by CD4	95% CI for each rate	Holmes [35]
Portion of the population ever tested for HIV, 2010				
	2005	30%		Rwanda DHS
	2010	65%		Rwanda DHS
	2013	78%		Nsanzimana [10]

### HIV disease, care, and mortality

Our model represents HIV disease and care along the care continuum. Untreated HIV disease is represented as a chronic progressive disease in which a declining CD4 count defines the risk of mortality and development of opportunistic diseases (OD). Effective treatment with ART leads to CD4 count increases that are based on age, CD4 count at treatment initiation, and duration of treatment. Additional details on the disease progression assumptions are in the Additional file 1 and previous articles [15, 16].

The flow of infected individuals through the HIV care system is characterized by the following stages. Individuals are unaware of their infection status prior to HIV testing (we call this stage Untested); we calibrated the annual rate of testing such that 33% of the adult population had been tested by 2005 and 71% by 2013. In 2013, 80% of those who tested positive were linked to care. We term those who are aware of their status and never linked to care Unlinked. During the period between initial linkage and ART initiation, patients are said to be pre-ART, and may become Leaked from care with a monthly probability of 0.5%. A corollary is that late treatment initiation could lead to increases in the number of leaked patients, who may re-present to HIV care only when they develop an OD [10]. Rwanda uses CD4 cell counts staging for treatment initiation, and in our analysis patients initiate ART when their measured CD4 count drops below 250 cells/mm3 (2003 to 2008), 350 cells/mm3 (2009–2013), or 500 cells/mm3 (starting in 2014). Patients also qualify for ART when they experience an OD. Given estimates of rates of transmitted drug resistance, we estimate that 90% of those who are adherent achieve viral suppression within 6 months of ART initiation [26, 27]. Following ART initiation, patients may be lost to follow-up (LTFU) with a monthly probability of 0.0875% [10]. We use estimates that 50% of those LTFU may return to care if they develop an OD [9, 10].

We used monthly mortality rates for each person as a combined function of age, gender, and, for HIV-positive individuals, CD4 count, and ART status. Age- and gender-specific mortality for HIV-negative individuals come from life tables for Rwanda and converted to monthly probability of death [28]. We cause-delete the contribution of HIV to overall mortality using estimates of the portion of deaths due to HIV by age and gender [29]. Thus, for HIV-negative individuals we apply cause-deleted age- and gender-specific mortality. For HIV-positive individuals, we model mortality as a function of current CD4 cell count, with an additional short-term risk of death during an acute OD event [30]. All the influential base case parameter and uncertainty values are summarized in Table 1.

### Outcomes and sensitivity analyses

Our principal analyses address the following two questions: (1) where are HIV-infected individuals dying in the context of Rwanda’s care continuum? and (2) how are these patterns expected to vary with changes to HIV care in Rwanda? In analyzing mortality, we measure deaths among HIV-infected individuals in each of the following groups: prior to HIV testing (Untested), among those with a known HIV diagnosis but who are no linked to care (Unlinked), after linkage to care but prior to ART initiation (pre-ART, included leaked patients), among those in ART care, and among those lost to follow-up (LTFU) after ART initiation. We estimate mortality with two metrics: the portion of all deaths taking place among individuals in each group, and a mortality rate calculated as the number of deaths divided by the number of person-years in the group (age-adjusted using weighted average of rates in each 5-year age group). The point estimates for the base case were obtained after 100 model iterations. Uncertainty in the results reflect both first-order (stochastic) variation as well as second-order variation from a probabilistic sensitivity analysis where parameters were jointly sampled from the distributions spanning their uncertainty ranges and the outcomes of interest were summarized after 1000 model iterations. Uncertainty bounds are reported as the 25–75% range among all model iterations.

In addition to the base-case estimations, we simulate the expected changes in mortality under several potential changes to Rwanda’s national strategic plan for HIV control. These include modeling the mortality changes following intensifying annual testing rates from 40% to 90% of the population; increasing linkage to care from 80% of the tested population to 90% (for example using patient tracing); reducing leakage from pre-ART 0.5% to 0.1% of the pre-ART population per month; and improving rates of LTFU from 0.0875% of those on ART to 0.05% per month. We also simulated immediate ART initiation for all linked HIV-positive individuals and one combined scenario where all improvements were implemented. These improvements were constructed to reflect proposed interventions in Rwanda through early identification and offer of ART to HIV-infected individuals, targeting of populations at high risk for infection, community support for retention in ART programs, and peer networks to improve maintenance in care and reduce rates of loss to follow-up.

All simulations were carried out in Matlab 2014b, and analyses and visualizations in Stata 13.1.

## Results

**Fig. 1:**
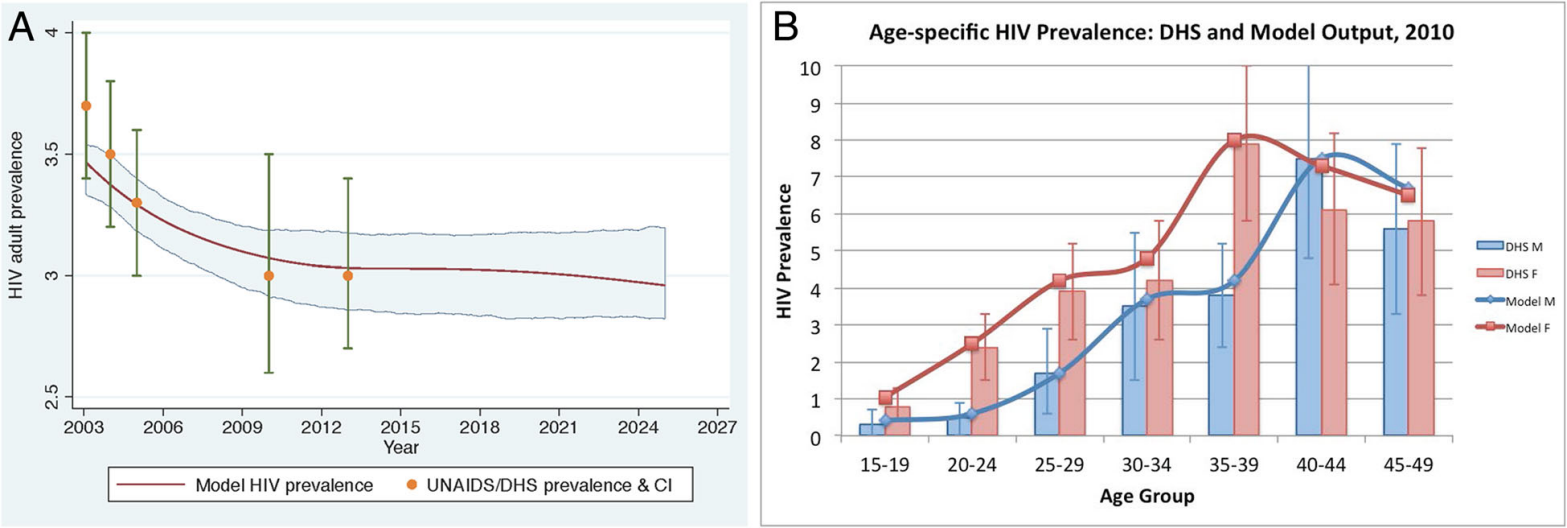
Panel 1**a** shows overall prevalence calibration (2003–2013) and projection (2014–2025). Dots and uncertainty bars represent prevalence estimates from UNAIDS (2003 and 2004) and DHS (2005, 2010, and 2013). Red line represents model prevalence, and the shaded area represents 25–75 percentile range from 100 model runs. Panel 1**b** shows calibration results of prevalence for 5-year age and gender groups. This figure shows that 7 years after the initial model setup, age- and gender-specific prevalence match estimates from the 2010 population-level DHS survey

Our model is calibrated to Rwanda’s overall and age-specific HIV prevalence from 2003 to 2013, and projected forward to 2025. Between 2003 and 2005, we calibrated the model to UNAIDS prevalence estimates among adults, declining from 3.6% in 2003 to 3.3% in 2005 [1]. From 2005 to 2013, we used the Rwanda Demographic and Health Surveys of 2005 and 2010, and the Rwanda AIDS Indicators Survey of 2013 to obtain nationally representative adult HIV prevalence estimates [21]. Adult HIV prevalence was 3.0% in all three surveys. Prevalence in our model declined to 3.0% in 2007, and remained stable during the period from 2007 to 2013. We calibrated overall prevalence as well as age-specific prevalence in each 5-year age group, available from the Rwanda Demographic and Health Surveys. Figure 1 shows the model’s calibration fitting overall prevalence estimates from 2003 to 2013 (panel A) as well as age-specific prevalence rates seven years into the model is started, in 2010 (panel B). Although we did not explicitly calibrate incidence, our model’s incidence for 2013–2014 was 0.23 infections per 100 person-years, similar to the 0.27 estimate in the 2013–14 Rwanda HIV Incidence Household Survey [31]. The Additional file 1 contains additional calibration results.

Several demographic trends shape the mortality patterns. First, a stable 3% adult HIV prevalence, combined with an overall annual population growth rate of 2.6% implies a steady growth in the HIV-positive population, estimated at 239,668 in 2014 and increasing 25.9% to 301,808 by the end of 2025. Over the same time period, a continuation of current HIV mortality rates imply an increase of 17.4% in the number of deaths among those HIV-positive, from 12,971 in 2014 to 15,229 in 2025. While these estimates represent substantial increases in HIV-infected populations and deaths, they are smaller than the projected 32.6% increase in the overall population size, suggesting a decline from the present in HIV burden. Our baseline estimates of the number of deaths are higher than UNAIDS estimates of AIDS deaths since the model accounts for all deaths among those with HIV, including non-AIDS deaths among those with HIV. This increase in the number of deaths among those with HIV reflects the balance between the upwards pressure on mortality from population growth and aging of the HIV-infected population versus the declining mortality from continued expansion of the population accessing ART. As Rwanda’s HIV-infected population ages, the portion of deaths made up by older individuals is also expected to increase. Our model estimates that, in 2014, 10% of deaths among HIV-infected individuals took place among those older than 65-years-old, and that this proportion would increase to 24% in 2025. These changes in HIV population patterns, including population growth and aging, help frame the changing drivers of mortality.

### Where are people dying along the care continuum?

**Fig. 2:**
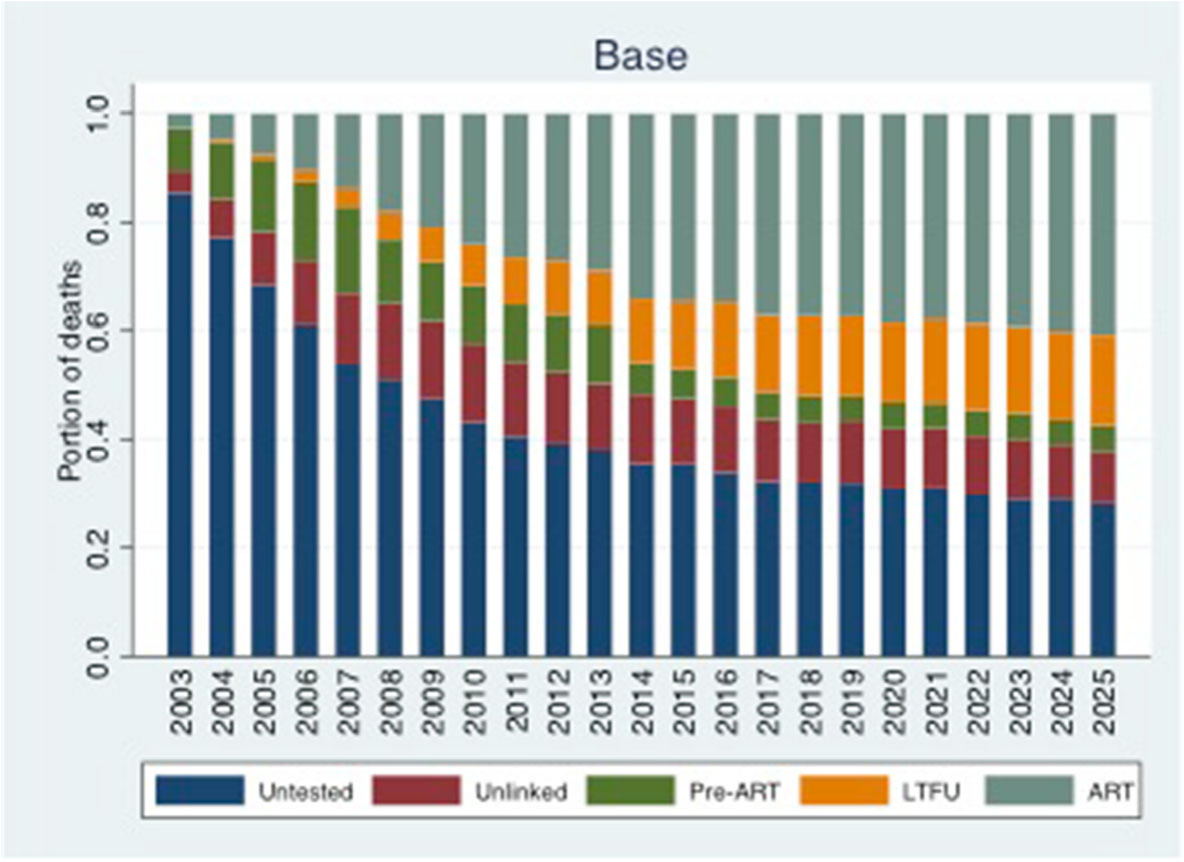
The proportion of deaths among people with HIV by stage of care. Mortality is high among those unlinked to care in the early years of treatment scale-up, as testing identifies many people with late-stage disease. Over time, the proportion of deaths among those unlinked declines and those LTFU increases. The portion of deaths among those on ART remains stable, though the mix of AIDS-related and non-AIDS-related deaths changes over time

Our base case mortality analysis suggests that in 2003, untested individuals accounted for around five-sixths of deaths among people with HIV (85.4% [80.1–90.5%] of an estimated 17,025 deaths), but that this proportion had declined with increasing testing coverage and the changing composition of those untested to include a growing portion of asymptomatic individuals with relatively higher CD4 counts. Figure 2 shows the time trends and changes in mortality distribution under the base case scenario. This results from selective testing of those with ODs such that the testing rate is generally higher among those with advanced disease, and from the opportunity to miss those with recent infections even when testing rates are high, leading to a shift in the untested population towards those with less advanced HIV (we estimate that among those untested, the portion with CD4 cell counts higher than 500 was 29.4% in 2003 and 59.3%in 2014). By 2014, the portion of deaths among those untested declined to 35.4% (31.2-39.5% out of an estimated 8,177 deaths among people with HIV). At the same time, those on ART experienced the largest growth in the proportion of deaths, from 2.7% in 2003 (1.1–4.4%) to 34.1% in 2014 (29.9–38.4%). This is a reflection of the large growth of people on ART, and accounts for the overall decline in mortality rates among those with HIV, since the mortality rate among those on ART is lower than any other group. We project that, over the next decade, the portion of all deaths made up by those untested, unlinked, and pre-ART will decline, and the portion of all deaths made up by those on ART and LTFU will increase (where deaths on ART is a desirable outcome of effective scale-up, while deaths among those lost from care are undesirable). The greatest increase in mortality is estimated to take place among those LTFU, from 11.8% (11.1–12.5%) in 2014 to 18.8% (17.9–19.9%) in 2025. While empirical observations and our modeled population suggest that in 2013 those LTFU made up 3% of the population living with HIV, model findings suggests that this group’s share of overall deaths is about 4 times higher than its share of the population, reflecting their high mortality rate, especially in comparison with those on ART [10]. The increasing mortality share of those LTFU is a reflection of the growing portion of the infected population initiating ART, and, at current rates of loss from care following ART initiation, the number of those lost from ART care.

**Fig. 3:**
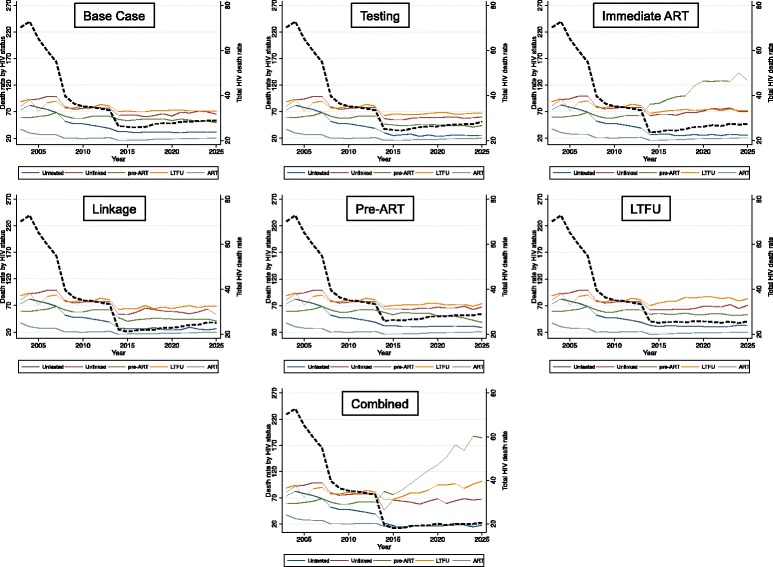
Mortality rate (defined as the number of deaths divided by 1000 person-years lived in the year) among people with HIV. The estimates are age-adjusted by providing an average of the mortality weighted by the proportion of individuals in each 5-year age group for each health state. Each panel contains overall HIV mortality rate (*dashed black line*, right-hand axis) and the mortality rate in different care continuum groups. The first panel represents the trends assuming base case level of care. Subsequent panels represent the trends with the indicated improvements to HIV care starting in 2014. A combined intervention with improvements to all aspects of HIV care is projected to lead to the greatest reduction in mortality rates

Mortality rates among all HIV-infected individuals have declined from a peak of 72.8 deaths per 1000 person-years in 2004 to 26.3 in 2014 (Fig. 3). These declines are mirrored most strongly in reductions among those untested and those on ART. Mortality rates among those unlinked, during pre-ART, or those LTFU have only declined modestly.

### Effects of targeted and combined care improvements

Because Rwanda’s HIV care system is currently functioning at a high level in terms of HIV testing, ART coverage, and retention in care, it’s baseline rate of mortality among people with HIV is relatively low. Whether this is more due to efficiency in the program or due to high levels of investments in HIV control, only combined improvements to HIV care are expected to lead to large reductions in mortality rates. Improvements to an isolated part of the care continuum yield reductions in the contribution to mortality from that stage of care, with concomitant increases in contributions to mortality from downstream stages of care. For example, an isolated effort to increase testing rates could reduce the contribution to mortality among the untested from 26.8% in 2013 to 3.8% in 2025, while increasing the contribution to mortality among those who are unlinked, in pre-ART, LTFU, or on ART by 4.2%–7.7% (Additional file 1: Section S3). A combined package of improving all aspects of HIV care is expected to reduce overall mortality rate among HIV-infected individuals to 19.9 (19.3–20.6) deaths per 1,000 person-years, resulting in a declining number of deaths among those with HIV to 5691 in 2025. In addition, a combined package of interventions is also expected to result in the greatest prevalence decline, down to 2.1% (2.0-2.3%) by 2025 (Additional file 1: Section S4).

## Discussion

Our analysis provides new insights into mortality among people with HIV in Rwanda, with potential implications for other countries in sub-Saharan Africa with similar demography and epidemiology. Four principal insights emerge: first, at the current performance of the HIV healthcare system, the combination of growing population size, aging, and stable HIV prevalence implies that the number of deaths among HIV-positive individuals are expected to increase; second, while the untested and on-ART populations currently make the greatest contributions to mortality, the current levels of testing and ART initiation are expected to result in a declining share of mortality from those untested and a growing share from those in ART care; third, among those not on ART, mortality among those LTFU is expected to grow the most over the next decade; and, fourth, a combined approach that includes improvements to all aspects of HIV care may be needed in order to produce meaningful mortality decline.

We believe our analysis is the first HIV transmission model calibrated specifically with Rwanda’s epidemiologic and treatment parameters. HIV care in Rwanda has been among the best in sub-Saharan Africa in terms of expanding ART coverage through investments in HIV care infrastructure, adoption of higher CD4 initiation thresholds, and improving HIV testing and linkage rates. At the same time, like several other sub-Saharan countries with rapidly growing ART programs, Rwanda had witnessed flat prevalence rates for much of the last decade and a declining HIV financial budget, such that maximizing care is a priority. In this complex landscape, our analysis identifies drivers of mortality along the care continuum.

Our modeling approach highlights the important role played by demographic changes. Specifically for Rwanda, population growth and aging of the overall and HIV-infected populations imply that growing numbers of individuals will die with HIV, often while remaining in ART and HIV care. On the one hand, this means that the growing mortality share of those on ART reflects the desirable survival of this population to older ages. On the other hand, it underscores the future needs to address age-related mortality and morbidity in this population [32].

The population growth and resulting increasing deaths in the base case scenario also highlight the long-term benefits of combined improvements to the cascade. While immediate ART initiation, as a stand-alone improvement, reduces mortality overall, those benefits are modest in comparison with a combined intervention over a 10-year period. In our model, immediate ART prevents leakage and reduces mortality among those in pre-ART, and secondarily reduces mortality through its effects on ongoing transmission and incidence, but that effect is modest over a 10-year time horizon. A combined strategy further reduces mortality among those untested, unlinked, and LTFU. However, implementing immediate ART may be substantially simpler than a combined strategy (similar to UNAIDS’s 90-90-90 targets), and may simplify HIV care by avoiding pre-ART care.

There are both strengths and limitations to our analysis. The strengths include our access to Rwandan specific data and interaction with the Ministry of Health officials available to provide context. Our model integrates a large amount of information on HIV epidemiology and care. Specifically, the mortality projections rely on data from observational and surveillance cohorts, and any bias in those parameters introduced by selection will propagate in our model. Our model assumes predominantly heterosexual transmission. Given stigma associated with men who have sex with men in Africa and a lack of data on injecting drug use, it is possible these populations play a larger role than we accounted for. In addition, while Rwanda’s population growth has remained stable over the past decade, our findings of the number of deaths could change if the demographic projections of population growth and age structure will change substantially over the next decade.

As with all model-based analyses, there are trade-offs regarding the choice of underlying data and modeling approach. We used a microsimulation modeling technique to capture the age and gender risk factors that are highly relevant to the key projections of mortality by disease stage. The microsimulation modeling approach offers important advantages over deterministic models, but the extensive data requirements limit the model’s generalizability. For instance, our model simulates HIV transmission in Rwanda through heterosexual contact, with heterogeneity in transmission represented only through the number of sexual partners. Key populations such as sex workers or injection drug users play an important role in the transmission network of HIV, but the dearth of data on their epidemiology and behavior limits our ability to incorporate them in the modeling framework.

## Conclusion

In conclusion, estimating the mortality patterns among those with HIV enables planning of interventions towards those groups that are expected to shoulder the burden. We estimate that the greatest increase in mortality among those not on ART is expected among those LTFU, and the greatest decline among those untested. However, we also show that interventions targeting a single part of the HIV care space will not meaningfully decrease the number of deaths among those with HIV, and that combined approaches may be required to continue shrinking the number of deaths among those with HIV.

## Additional file

Additional file 1:
**Section S1.** Additional Model Schematic, Description, and Parameters. **Section S2.** Model Calibration Figures. **Section S3.** Changing Mortality Distribution with Continuum Improvements. Section S4: Projected Prevalence Trends with Continuum Improvements. (DOCX 12869 kb)

## Abbreviations

ART: Antiretroviral therapy
HIV: Human Immunodeficiency virus
LTFU: Lost to follow up
UNAIDS: The joint United Nations programme on HIV/AIDS
