# Supplementary material for: Mortality along the continuum of HIV care in Rwanda: a model-based analysis
Source: BMC Infect Dis. 2016 Dec 1;16:728. doi: 10.1186/s12879-016-2052-7 (PMC5134104; doi:10.1186/s12879-016-2052-7)
Supplement: Additional file 1: — Section S1. Additional Model Schematic, Description, and Parameters. Section S2. Model Calibration Figures. Section S3. Changing Mortality Distribution with Continuum Improvements. Section S4: Projected Prevalence Trends with Continuum Improvements. (DOCX 12869 kb) [file 12879_2016_2052_MOESM1_ESM.docx]

Supplementary Appendix for “Mortality Along the Continuum of HIV Care in Rwanda: A Model-Based Analysis”

Eran Bendavid, David Stauffer, Eric Remera^,^ Edward Mills, Steve Kanters , Sabin Nsanzimana

The Supplementary Appendix contains the following sections:

Section S1: Additional Model Schematic, Description, and Parameters 2

Section S2: Model Calibration Figures 10

Section S3: Changing Mortality Distribution with Continuum Improvements 14

Section S4: Projected Prevalence Trends with Continuum Improvements 18

References 21

Corresponding author: Eran Bendavid MD MS, Division of General Medical Disciplines, Stanford University, Stanford CA 94305. Telephone: +1-650-723-2363, Fax: +1-650-723-8596, email: [ebd@stanford.edu](mailto:ebd@stanford.edu).

# Section S1: Additional Model Schematic, Description, and Parameters

*Model Schematic Figure*

The schematic above represents two general dimensions of the model. One dimension tracks the history and transition of individuals through different states in the HIV care continuum (top portion), and the second dimension tracks individuals’ HIV disease progress. They are two dimensions in the sense that patients can (largely) progress along each dimension at each health state of the other dimension. For example, the HIV disease model progresses for individuals who are on 1^st^ line ART differently than for those who are lost to follow-up (LTFU). The dashed arrows out of each box indicate the possibility of death conditional on the health state, HIV stage, and demographic characteristics. The diagonal arrows leading to ovals represent the pathways patients may end up being “off care.” The names of the “off care states in the ovals correspond to the names used in the manuscript. The model description below provides more details on the assumptions underpinning patient progress.

*Model Assumptions and Description*

General population: Every iteration of the model explicitly tracks 1,000,000 individuals that are stratified to look like the gender and age composition of Rwanda’s population ages 15 and older. Age is distributed according to population pyramids and population growth is set to the projected population growth for the entire population of Rwanda, which may or may not differ from the population growth rate of the 15+ population. Age is tracked monthly.

Each age-gender group has a general non-HIV (“background”) mortality rate that reflects causes of death such as non-communicable diseases, accidents, and most communicable diseases. We did not model any interactions between HIV and other causes of death except for opportunistic infections (including TB), so that the background mortality rate is similar for HIV-positive and HIV-negative individuals of the same age-gender group. The background rate of death was anchored in information available in life tables. However, in recognition of the facts that life tables use information for the entire population (HIV-positive and HIV-negative), and that HIV makes up a meaningful component of mortality among young adults, we estimated the contribution of HIV to mortality in the overall mortality rate and extracted that component from the mortality rates. We did this estimation by extracting the age- and gender-specific portion of deaths from HIV as indicated in the Global Burden of Disease, and reduced the corresponding mortality rate by that amount. That led to greater reductions in background mortality for young women and men during the ages of peak HIV burden than in older women and men.

The general population was further characterized by features that enabled estimation of HIV transmission risk, as noted in the Methods section. These include the number of sexual partners, and, for men, their circumcision status.

HIV Care Model – States and Transitions: Following HIV infection and a 2-month period of acute HIV, patients are characterized by the following staged of HIV care.

- “Untested” consists of two states:

1. Undiagnosed: All HIV-infected individuals are in this state before being tested and diagnosed. No one in this state receives CD4 testing or ART, and CD4 cell counts decline on average at a rate related to the viral load. The individuals in this state are tested at a rate that corresponds to the national HIV testing rate and matches the portion of the population reporting having been tested for HIV (calibration figure in S2 below).
2. Undiagnosed “off care”: this population represents the portion of individuals that remain untested even when the supply of testing through national programs or NGOs is abundant. This represents the upper bound of testing capacity and is taken from observations of the greatest achievable testing coverage in national and sub-national populations. In Rwanda the undiagnosed “off care” population ranged from 20% in 2005 to 5% in 2015 (upper bound on the diagnosed population 80% to 95%).

- Unlinked “off care”: in this state individuals who have been given their HIV+ status never connect with an HIV clinic, never receive staging CD4 cell counts, and do not initiate ART based on CD4 threshold criteria. Half of these individuals could transition into care upon the development of acute OD.
- Diagnosed, linked, pre-ART (pre-ART): in this state individuals have been diagnosed and have received at least one CD4 count but are considered ineligible for ART because of their high CD4 cell count. This was a substantial group when CD4 cell count thresholds for ART initiation were 250 or 350 cells/mm^3^. It is diminishing with higher initiation thresholds, and earlier initiation of ART.
- Leaked “off care”: this state includes individuals who had been in pre-ART care but had fallen out of care in the period of time while waiting for ART initiation. The rate of loss of individuals through this route is described in the parameter tables. Since the loss from care into this state is rate-dependent, shortening the pre-ART period reduces the opportunity to leak from HIV care and diminishes the size of this state. In main manuscript, the Leaked state and the pre-ART state were merged into a single state for mortality purposes.
- First- and second-line ART: in these states individuals receive NNRTI-based or PI-based ART, respectively, and the effectiveness in terms of rates of virologic suppression and CD4 cell count changes is guided by observed data. Starting in 2010, transition from first to second-line ART was determined by monitoring viral load and identifying virologic failure on 2 consecutive tests.
- Loss to follow-up: in this state individuals stop receiving (and taking) ART, and as a consequence their viral load climbs, CD4 cell count declines, and their risk of developing ODs and dying reflect their off-ART state. Since this is a rate-dependent event, the longer the expected duration of ART (that is, the earlier ART is initiated), the more this group will be expected to grow. Half of these individuals could transition into care upon the development of acute OD.

HIV Care Interventions – Assumptions and Changing Dynamics:

The following HIV care improvements were simulated to examine the projected changes in mortality patterns:

- Testing: In this scenario, annual testing rates increased from 40% of the population to 90% of the population. Recall that there is an upper bound on testing rates, which in Rwanda is 95%, so an annual 90% testing rate means that nearly all of the testing-eligible population is tested nearly annually. As seen in section S3 below, this leads to near-elimination of mortality among those untested. Increasing testing rates is a goal of Rwanda’s current HIV National Strategic Plan.[[1](#_ENREF_1)]
- Linkage: The focus of this improvement goal is to reduce the population that is diagnosed with HIV but not linked to care. In practice this involved increasing the linkage to care from 80% of those diagnosed with HIV to 90%, as could possibly be achieved using patient tracing and follow-up with newly identified HIV-positive individuals. In this strategy, the portion of individuals who died while unlinked declined substantially (section S3 below), and the overall mortality rate declined more than in any other single strategy aiming to bring back patients who have been lost from care.
- Pre-ART: In this scenario, the portion of those linked and awaiting ART initiation that were lost from care declined from 0.5% of the population to 0.1%.
- Loss-to-Follow-Up (LTFU): In this strategy the monthly rate of loss from HIV care declined from 0.0875% of those on ART to 0.05% of those on ART. Other than the Combined strategy, this was the only strategy that reduced the relative portion of deaths among those who are LTFU.
- Immediate ART: This projection shows the potential implications of placing any linked HIV+ individual on ART irrespective of their CD4 cell count. As shown in Section S3 below, this nearly eliminates the mortality among those in the pre-ART stage, since in this strategy individuals are not waiting for ART initiation, and do not linger in care or else are leaked from care. The mortality rate among those in pre-ART increases, since the population remaining in the pre-ART stage is increasingly composed of those who are lost from care in the very narrow time period between getting connected to services and initiating ART. That is, the only remaining individuals in this population are not in care.
- Combined: This situation includes all the above improvements to HIV care, and as a result a massive shift of individuals onto ART (and retention in care).

*Model parameters and uncertainty:*

| **Variable** | | **Base Estimate** | | **Range** | | | **Source** | |
| --- | --- | --- | --- | --- | --- | --- | --- | --- |
| *Demographics* | |  | |  | | |  | |
| Age distribution (% of population) | |  | |  | | | UN World Population Prospects[[2](#_ENREF_2), [3](#_ENREF_3)] | |
| 15-24 | | 33.2% | | ±5% | | |  | |
| 25-34 | | 26.2% | | ±5% | | |  | |
| 35-44 | | 19.2% | | ±5% | | |  | |
| >44 | | 21.3% | | ±5% | | |  | |
| Age-specific population mortality rate | | From Rwanda life tables | |  | | | Global Health Observatory[[4](#_ENREF_4)] | |
| Partnership distribution (# of annual sexual partners) | |  | |  | | | Rwanda DHS[[5](#_ENREF_5)] | |
| 0 partners | | 36% | | ±10% | | |  | |
| 1 partner | | 56% | | ±10% | | |  | |
| 2 partners | | 7% | | ±1% | | |  | |
| 3 partners | | 1% | | ±0.5% | | |  | |
| Proportion circumcised | | 30% | | ±5% | | |  | |
|  | |  | |  | | |  | |
| *HIV Transmission Parameters* | |  | |  | | |  | |
| Age-specific prevalence (mean for males and females) | |  | |  | | |  | |
| 15-24 | | 1.0% | | ± 0.5% | | |  | |
| 25-34 | | 3.3% | | ± 0.5% | | |  | |
| 35-44 | | 6.3% | | ± 0.5% | | |  | |
| >44 | | 3.0% | | ± 0.5% | | |  | |
| Risk of infection per sex act, by log viral load | |  | |  | | | Wawer, Gray, Quinn[[6-8](#_ENREF_6)] | |
| <2.7 | | 0.0001 | | 0-4x | | |  | |
| 2.7-3.48 | | 0.0011 | | 0.5-2x | | |  | |
| 3.49-4 | | 0.0012 | | 0.5-2x | | |  | |
| 4.01-4.48 | | 0.0014 | | 0.5-2x | | |  | |
| >4.48 | | 0.0023 | | 0.5-2x | | |  | |
| Late stage AIDS | | 0.0043 | | 0.5-4x | | |  | |
| Acute infection | | 0.0081 | | 0.5-4x | | |  | |
| Circumcision risk reduction | | 50% | | 40%-60% | | | Bailey, Auvert, Gray[[9-11](#_ENREF_9)] | |
|  | |  | |  | | |  | |
| *HIV Disease Parameters* | |  | |  | | |  | |
| Mean and standard deviation viral load in untreated (log_10_) | | 4.8 (0.85) | |  | | |  | |
| Monthly probability of developing opportunistic diseases (%), by CD4 | *<50 cells/μl* | | *51-200 cells/μl* | | *201-350 cells/μl* | *>350 cells/μl* | |  |
| Oral candidiasis | 3.50% | | 2.04% | | 1.26% | 0.57% | |  |
| Chronic diarrhea | 2.00% | | 0.49% | | 0.18% | 0.00% | |  |
| Esophageal candidiasis | 1.46% | | 0.34% | | 0.09% | 0.06% | |  |
| Wasting syndrome | 1.29% | | 0.23% | | 0.02% | 0.00% | |  |
| Severe bacterial infection | 1.15% | | 0.04% | | 0.03% | 0.00% | |  |
| Pulmonary TB | 0.85% | | 0.51% | | 0.37% | 0.11% | |  |
| Extrapulmonary TB | 0.48% | | 0.27% | | 0.08% | 0.03% | |  |
| PCP | 0.67% | | 0.05% | | 0.02% | 0.00% | |  |
| CMV | 0.52% | | 0.07% | | 0.02% | 0.00% | |  |
| Cryptococcal meningitis | 0.52% | | 0.05% | | 0.00% | 0.00% | |  |
| Monthly HIV mortality without ART, by CD4 count, 2003 (improving over time) | |  | |  | | |  | |
| 0-49 | | 4.8% | | 0.5-2x | | |  | |
| 50-99 | | 1.9% | | 0.5-2x | | |  | |
| 100-199 | | 1.5% | | 0.5-2x | | |  | |
| 200-299 | | 1.2% | | 0.5-2x | | |  | |
| 300-399 | | 1.0% | | 0.5-2x | | |  | |
| 400-499 | | 0.8% | | 0.5-2x | | |  | |
| ≥500 | | 0.5% | | 0.5-2x | | |  | |
| Monthly HIV mortality with ART, by CD4 count, 2003 (improving over time) | |  | |  | | |  | |
| 0-49 | | 3.2% | | 0.5-2x | | |  | |
| 50-99 | | 1.1% | | 0.5-2x | | |  | |
| 100-199 | | 0.4% | | 0.5-2x | | |  | |
| 200-299 | | 0.2% | | 0.5-2x | | |  | |
| 300-399 | | 0.2% | | 0.5-2x | | |  | |
| 400-499 | | 0.2% | | 0.5-2x | | |  | |
| ≥500 | | 0.1% | | 0.5-2x | | |  | |

# Section S2: Model Calibration Figures

Population size:

Model predictions of population growth in Rwanda. Population growth is about 2.7% per year, and fits the estimated population size in Rwanda from 2003 to 2015 from the World Bank’s World Development Indicators.

Portion of HIV+ population ever tested for HIV:

Portion of people living with HIV that are on ART:

Model predictions of ART coverage for Rwanda compared with UNAIDS and World Bank estimates of coverage for the period 2004 to 2014. The curve flattens because of imperfect testing and linkage to care in the base case. The model jumps represent entry of individuals into ART during years when eligibility guidelines change.

HIV prevalence with forward projections in the base case, and calibration estimates and uncertainty bounds from UNAIDS models and DHS surveys.

# Section S3: Changing Mortality Distribution with Continuum Improvements

# Section S4: Projected Prevalence Trends with Continuum Improvements

# References

1. **Rwanda HIV and AIDS National Strategic Plan** [<http://rbc.gov.rw/IMG/pdf/final_nsp_2013-2018.pdf>]

2. **UN Population Division. World Population Prospects: The 2012 Revision** [<http://esa.un.org/wpp/>]

3. **World Population Prospects, the 2015 Revision** [<http://esa.un.org/unpd/wpp/>]

4. **Global Health Observatory Data Repository: Rwanda Life Tables** [<http://apps.who.int/gho/data/?theme=main&vid=61370>]

5. **National Institute of Statistics of Rwanda (NISR) [Rwanda], Ministry of Health (MOH) [Rwanda], and ICF International. Rwanda Demographic and Health Survey 2010.** In*.* Calverton, Maryland, U.S.A.: NISR, MOH, and ICF International; 2012.

6. Gray RH, Wawer MJ, Brookmeyer R, Sewankambo NK, Serwadda D, Wabwire-Mangen F, Lutalo T, Li X, vanCott T, Quinn TC: **Probability of HIV-1 transmission per coital act in monogamous, heterosexual, HIV-1-discordant couples in Rakai, Uganda**. *Lancet* 2001, **357**(9263):1149-1153.

7. Wawer MJ, Gray RH, Sewankambo NK, Serwadda D, Li X, Laeyendecker O, Kiwanuka N, Kigozi G, Kiddugavu M, Lutalo T *et al*: **Rates of HIV-1 transmission per coital act, by stage of HIV-1 infection, in Rakai, Uganda**. *J Infect Dis* 2005, **191**(9):1403-1409.

8. Quinn TC, Wawer MJ, Sewankambo N, Serwadda D, Li C, Wabwire-Mangen F, Meehan MO, Lutalo T, Gray RH: **Viral load and heterosexual transmission of human immunodeficiency virus type 1. Rakai Project Study Group**. *N Engl J Med* 2000, **342**(13):921-929.

9. Gray RH, Kigozi G, Serwadda D, Makumbi F, Watya S, Nalugoda F, Kiwanuka N, Moulton LH, Chaudhary MA, Chen MZ *et al*: **Male circumcision for HIV prevention in men in Rakai, Uganda: a randomised trial**. *Lancet* 2007, **369**(9562):657-666.

10. Auvert B, Taljaard D, Lagarde E, Sobngwi-Tambekou J, Sitta R, Puren A: **Randomized, controlled intervention trial of male circumcision for reduction of HIV infection risk: the ANRS 1265 Trial**. *PLoS Med* 2005, **2**(11):e298.

11. Bailey RC, Moses S, Parker CB, Agot K, Maclean I, Krieger JN, Williams CF, Campbell RT, Ndinya-Achola JO: **Male circumcision for HIV prevention in young men in Kisumu, Kenya: a randomised controlled trial**. *The lancet* 2007, **369**(9562):643-656.
